# Cxcr2 signaling and the microbiome suppress inflammation, bile duct injury, and the phenotype of experimental biliary atresia

**DOI:** 10.1371/journal.pone.0182089

**Published:** 2017-08-01

**Authors:** Junbae Jee, Reena Mourya, Pranavkumar Shivakumar, Lin Fei, Michael Wagner, Jorge A. Bezerra

**Affiliations:** 1 Divisions of Gastroenterology, Hepatology and Nutrition, Cincinnati Children’s Hospital Medical Center, Cincinnati, Ohio, United States of America; 2 Division of Biostatistics and Epidemiology, Cincinnati Children’s Hospital Medical Center, Cincinnati, Ohio, United States of America; 3 Division of Biomedical Informatics, Cincinnati Children’s Hospital Medical Center, Cincinnati, Ohio, United States of America; Texas A&M University, UNITED STATES

## Abstract

Biliary atresia is progressive fibro-inflammatory cholangiopathy of young children. Central to pathogenic mechanisms of injury is the tissue targeting by the innate and adaptive immune cells. Among these cells, neutrophils and the IL-8/Cxcl-8 signaling via its Cxcr2 receptor have been linked to bile duct injury. Here, we aimed to investigate whether the intestinal microbiome modulates Cxcr2-dependent bile duct injury and obstruction. Adult wild-type (WT) and *Cxcr2*^*-/-*^ mice were fed a diet supplemented with sulfamethoxazole/trimethoprim (SMZ/TMP) during pregnancy and lactation, and their pups were injected intraperitoneally with rhesus rotavirus (RRV) within 24 hours of life to induce experimental biliary atresia. The maternal exposure to SMZ/TMP significantly lowered the incidence of jaundice and bile duct obstruction and resulted in improved survival, especially in *Cxcr2*^*-/-*^ mice. Analyses of the microbiome by deep sequencing of 16S rRNA of the neonatal colon showed a delay in bacterial colonization of WT mice induced by SMZ/TMP, with a notable switch from *Proteobacteria* to *Firmicutes*. Interestingly, the genetic inactivation of *Cxcr2* alone produced a similar bacterial shift. When treated with SMZ/TMP, *Cxcr2*^*-/-*^ mice infected with RRV to induce experimental biliary atresia showed further enrichment of *Corynebacterium*, *Anaerococcus* and *Streptococcus*. Among these, *Anaerococcus lactolyticus* was significantly associated with a suppression of biliary injury, cholestasis, and survivability. These results suggest that the postnatal development of the intestinal microbiota is an important susceptibility factor for experimental biliary atresia.

## Introduction

Biliary atresia is a fibro-inflammatory process that disrupts the epithelium of the extrahepatic bile duct (EHBD), obstructs its lumen, and produces obstruction of bile flow early in the postnatal period [1, 2]. Even though the etiology of biliary atresia is multifactorial, the innate and adaptive immune systems have been shown to directly regulate early phases of epithelial injury and the biological continuum that underlies the pathogenesis of disease [3]. For example, IL-15 from rhesus rotavirus (RRV)-primed pDCs activates hepatic NK and/or T cells, resulting in injury of cholangiocyte and discontinuity of EHBD [4]. The cytotoxic effects of NK and CD8^+^ T cells are mediated by the release of soluble factors, such as perforin and granzymes [5]. In addition, the hepatic *IL8/Cxcl8* gene expression is increased at the time of diagnosis of biliary atresia above the levels seen in age-matched subjects with other causes of neonatal cholestasis [6]. A previous report showed that the release of IL-8/Cxcl-8 by macrophages attracts neutrophils to the site of biliary injury [7], and these cells have been linked to the intestinal colonization by commensals in neonates [8].

After birth, the neonatal intestine immediately faces dynamic changes as it transitions to a microbial-rich extra-uterine environment. Dysregulation of the early bacterial colonization or their composition in the intestine has been associated with immune-mediated pediatric diseases [9], and the function of pDC, T cells and NK cells can be substantially influenced by bacteria or their derivatives. For example, Polysaccharide A from *Bacteroides fragillis* suppresses inflammation by inducing the expression of IL-10 by regulatory T (Treg) cells [10]. The exposure of pDCs to polysaccharide A results in lower expression of TNF-α and IL-12/IL-23, and drives the differentiation of CD4^+^ T cells into IL-10-secreting cells [11]. As a whole, the microbiome has been shown to change the transcriptional profile of intestinal NK cells, significantly reducing transcriptional levels of IL-22 and RORγt in germ-free mice, suggesting that gut-microbiota regulate NK cell differentiation or effector function [12, 13]. Although pDCs, NK cells, T lymphocytes, and neutrophils have also been shown to play key effector roles in pathogenesis of biliary atresia, the role of the intestinal microbiome remains largely unexplored.

Based on our previous report linking IL-8/Cxcl-8 to pathogenesis of biliary atresia and the role of neutrophils in modulating the colonization of the intestinal microbiota in neonates, we hypothesized that the relationship between IL-8/Cxcl-8 signals and the neonatal microbiota modulates the phenotypic expression of experimental biliary atresia. We found that maternally administered antibiotics vertically affect the development of the gut-microbiome of neonatal Balb/c mice and protect them from the development of experimental biliary atresia in mice that lack a functional Cxcr2. The suppression of the disease phenotype was associated with a preferential abundance of *Anaerococcus lactolyticus*, raising the possibility that specific microorganisms or their metabolites may be involved in the pathogenesis of biliary atresia.

## Materials and methods

### Animals and antibiotic treatment

This study was carried out in strict accordance with the recommendations in the Guide for the Care and Use of Laboratory Animals of the National Institutes of Health. All animal protocols (IACUC2013-0151) were approved by the Institutional Animal Care and Use Committee of Cincinnati Children’s Hospital Medical Center. All experimental mice were maintained under humane conditions to minimize suffering and distress and in consultation with and supervision by veterinary staff. Wild-type (WT) BALB/c and *Cxcr2*^*-/-*^ mice in the BALB/c background (C.129S2[B6]-Cxcr2^tm1Mwm/J^) were acclimated and maintained in a specific pathogen-free facility with a 12 hour dark light cycle. To change the intestinal microbiome in neonatal mice, adult female and male mice were fed *ad libitum* separately on chow containing 0.03% of sulfamethoxazole (SMZ) and 0.14% of trimethoprim (TMP) (referred to as SMZ/TMP diet; TestDiet, Saint Louis, MO) for 4 weeks, and then paired for breeding and continued under the same antibiotic regimen throughout pregnancy. All dams and offspring were co-housed, and the mother continued to receive the SMZ/TMP diet through weaning.

### Experimental biliary atresia

Neonatal mice were injected intraperitoneally with 1.5 x 10^6^ fluorescence focus units (FFU) of RRV in a 20μl volume within 24 hours of birth as described previously [14]; 20μl of 0.9% saline solution was injected into additional litters to serve as controls. Mice were monitored daily for the development of jaundice, acholic stools, growth profiles, and mortality during the first 3 weeks of life. Most RRV-infected mice died within three weeks without intervention; moribund or mice with abnormal behaviors, such as inactivity, self-mutilation, cannibalism by littermates or lack of responsiveness, were euthanized following humane endpoints. No analgesics or anesthetics were used to minimize suffering in order to keep animals in the experimental design. At the time of organ harvest, mice were sacrificed using CO_2_ asphyxiation and blood exsanguination following IACUC-approved protocols. No mortality was observed before the study endpoint.

### Taxonomic microbiota analysis

At 3 and 10 days of life, mice were sacrificed by CO_2_ asphyxiation, followed by blood exsanguination to harvest colons and stored at -80°C until use. Total genomic DNA was extracted from the colons using QIAamp DNA Stool Mini Kit (Qiagen, Valencia, CA). Using the extracted DNA, the V4 region of 16S rRNA was amplified and sequenced by next-generation sequencing using Illumina MiSeq platform (Illumina, San Diego, CA), resulting in de-multiplexed 175 bp paired-end reads in each direction [15, 16]. We used a combination of best-of-breed analysis packages, including PANDAseq v2.8 [17], QIIME v1.8 [18] and USEARCH v7.0.1090 [19] along with custom scripts, and implemented a LONI [USC Laboratory of Neuro Imaging [20]] pipeline workflow for all 16S rRNA pre-processing steps to obtain OTU tables (S1 Fig). The paired-end reads from each direction were assembled to single sequences, resulting in 2,305,226 total reads (200 bp in minimum, 253 bp in median), and 1,002,813 sequences after dereplication. Sort-by-size, OTU clustering and mapping reads to OTUs resulted in 823 OTU representative sequences (935 OTU in minimum, 14124 OTU in median counts per sample). Taxonomic classification was generated using GreenGenes V13.8 database predefined taxonomy map of reference sequence at ≥ 97% similarity.

### Gene expression analysis

To quantify hepatic mRNA expression, we performed real-time RT-PCR using livers harvested 13 or 14 days after RRV-injection. The extraction and quality control of total RNAs, and amplification of the target genes were performed according to methods described previously [21]. The target genes were selected on the basis of protein-network with CXCR2 by using the STRING database (https://string-db.org/) and included *Cxcl1*, *Cxcl2*, *Cxcl3*, *Cxcl5*, *Cxcl15*, *Tnf*, *Cxcl10*, *Ccr1*, *Ccr2*, *Ccl2* and *Ccl12*. Sequences of primer sets are listed in S1 Table.

### Nested PCR

To identify species level of *Anaerococcus*, the extracted genomic DNA was pooled and amplified with concentrated FastStart PCR Master mix (Roche, Indianapolis, IN) and sets of primers listed in the S2 Table. First, 16S rRNA was amplified by primers 1 and 2, and the amplicon was analyzed by electrophoresis using 1% agarose gel. The 1500 bp amplicon was extracted from the gel and used as a template to screen for 6 species of *Anaerococcus* using primer pair combinations outlined in the S2 Table.

### Histopathology

At 14 days after RRV infection, mice were sacrificed to harvest EHBDs and livers, which were fixed with 10% formalin for 24 hours, embedded in paraffin, cut in longitudinal sections, stained with hematoxylin and eosin (H&E), and examined under an Olympus BX51 microscope and cellSens Dimension digital imaging software (Olympus America Inc., Center Valley, PA).

### Statistical analysis

Generalized linear model with logit link was applied to binary responses of jaundice (Y/N), associated with two variables, *i*.*e*., treatment group (regular- or SMZ/TMP-WT, and regular- or SMZ/TMP-*Cxcr2*^*-/-*^ mice) and days after RRV-injection. Then, Wald test was used to test significant relationship between logit of jaundice rate and each independent variable. For pairwise comparison of jaundice rates among the four treatments across all study days, equality of coefficients from two different regression analyses was tested using Wald test with Bonferroni correction. Kaplan-Meier curves were generated to depict survival probability in the first 3 weeks of life, and Log-rank test with Bonferroni correction was used for multiple comparisons of the survival curves. Fisher’s exact test was used to test if day of life, RRV-infection, treatment or mouse strains significantly affected bacterial colonization in 2 x 2 contingency tables. In addition, relative risk was used to see the strength between the two categorical data. Alpha diversity (within groups) was characterized by richness using observed taxa and Chao 1, and by evenness using Dominance and Shannon diversity index. For beta diversity (between groups), non-metric dimensional scaling (NMDS) was used to ordinate the microbial communities based upon Bray-Curtis similarity. The stress values were shown in each NMDS plot to indicate goodness of fit. Analysis of similarity (ANOSIM) with 9999 permutations was used to test statistical significance for pairwise comparisons among the bacterial communities. Significantly different taxa between regular and SMZ/TMP diets in each mouse were identified by linear discriminant analysis (LDA) effect size (LEfSe) with 3.5 of threshold on the logarithmic score of LDA analysis [22]. Circular cladograms showing the significantly different taxa were generated by GraPhlAn [23]. Kruskal-Walis ANOVA, followed by Dunn’s multiple comparison test was used to test significant differences in alpha-diversity and phylum-level of analysis. Mann-Whitney U test was used to examine significant differences in bacterial signature or hepatic mRNA levels between regular- and SMZ/TMP-diet or between diseased and resistant phenotype. Classification and regression tree (CART) was used to identify bacteria and estimate the frequency of the bacteria that clearly discriminate the dichotomous survival outcomes. In the CART analysis, frequencies of *Corynebacterium*, *Anaerococcus* and *Streptococcus* genus and dichotomous categorical outcomes, *i*.*e*., low survival that recoded survivability from regular-WT, SMZ/TMP-WT and regular-*Cxcr2*^*-/-*^ mice; high survival that recoded survivability from SMZ/TMP-*Cxcr2*^*-/-*^ mice were analyzed without information of mouse type and diet. Statistical analyses were performed with STATISTICA 7 (StatSoft, Tulsa, OK), Prism 6 (GraphPad Software, San Diego, CA), SAS 9.3 (SAS Institute, Cary, NC) and PAST 3 software [24].

## Results

### SMZ/TMP and loss of *Cxcr2* improve disease phenotypes in RRV-infected neonatal mice

**Fig 1:**
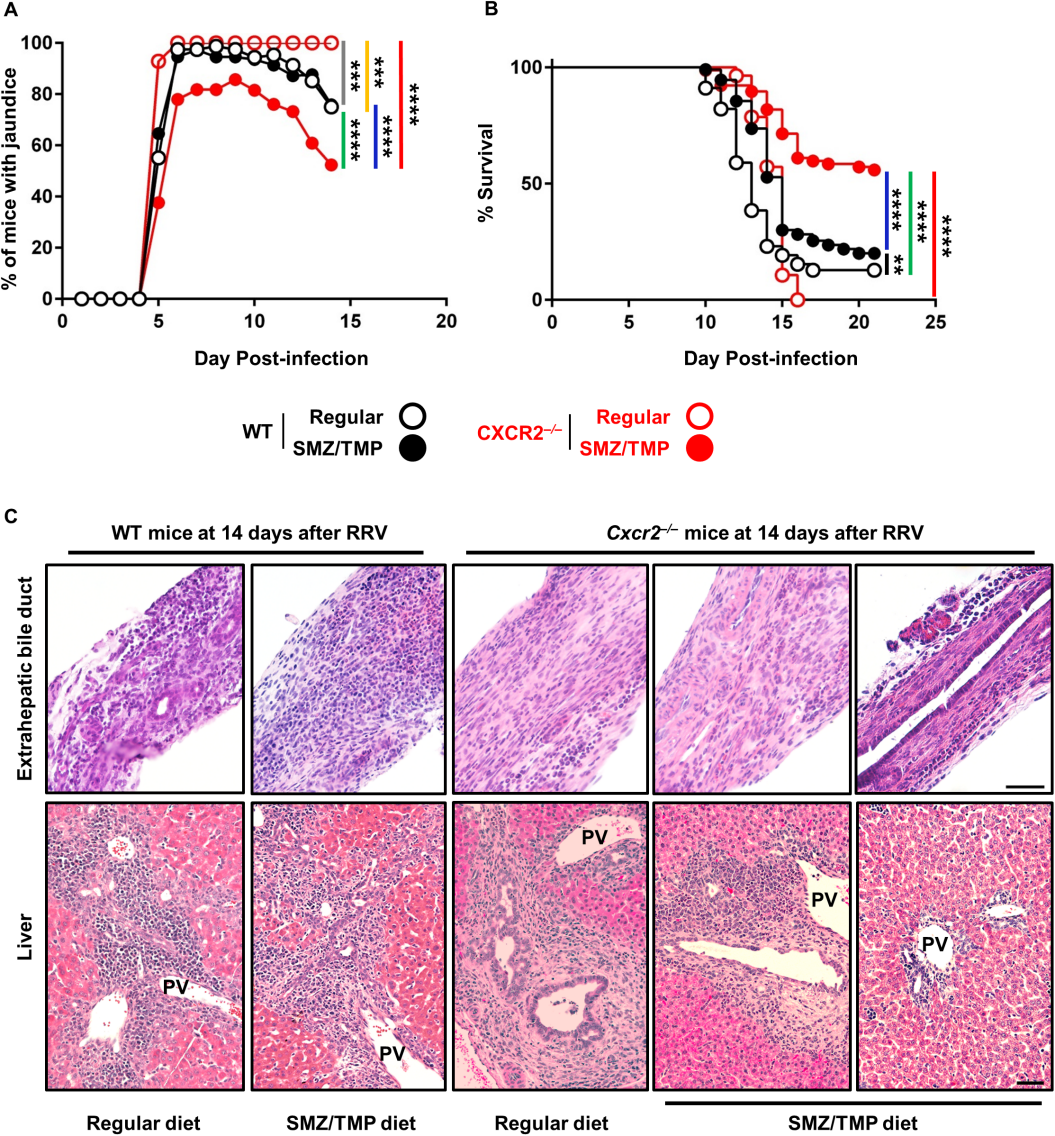
Clinical and tissue phenotypes in the SMZ/TMP-treated WT and Cxcr2^-/-^ mice. (A) Development of jaundice and (B) survivability were examined following RRV-injection (N = 28–110 per group). Black bar: comparison between regular-WT and SMZ/TMP-WT; yellow bar: regular-WT and regular-Cxcr2^-/-^; green bar, regular-WT and SMZ/TMP- Cxcr2^-/-^; gray bar, SMZ/TMP-WT and regular-Cxcr2^-/-^; blue bar, SMZ/TMP-WT and SMZ/TMP-Cxcr2^-/-^. *, P<0.05; **, P<0.01; ***, P<0.001; ****, P<0.0001. (C) Longitudinal sections of EHBDs and liver sections stained with H&E from the SMZ/TMP-treated Cxcr2^-/-^ mice. Unobstructed lumen and improved portal inflammation are observed in those Cxcr2^-/-^ mice alive at 14 days after RRV-injection (N = 4–5 per group); magnified with x20 (bar = 50μm).

To investigate the effect of altered gut-microbiome on experimental biliary atresia, adult mice were treated with SMZ/TMP and their offspring were injected with RRV as reported previously [14, 25]. In WT mice, SMZ/TMP did not change the onset of jaundice at 5 days after injection or thereafter (Fig 1A), but induced a small but statistically significant increase in survival above regular diet (12.7% in regular vs. 19.1% in SMZ/TMP, P < 0.01, Fig 1B).

To examine the potential modulation by IL-8/Cxcl-8, adult and newborn *Cxcr2*^*-/-*^ mice were subjected to the same experimental protocol. The frequency of jaundice was lower in neonatal *Cxcr2*^*-/-*^ mice 14 days after RRV when their mothers received SMZ/TMP (100% in regular diet vs. 52.4% in SMZ/TMP, P < 0.0001; Fig 1A), along with improved survival when compared to regular diet (0% in regular diet vs. 55.8% in SMZ/TMP, P < 0.0001; Fig 1B). Analysis of EHBDs and livers 14 days after RRV showed two histological patterns in SMZ/TMP-*Cxcr2*^-/-^ mice: 1) findings similar to WT mice of obstructed EHBD and expanded portal tracts with inflammation when they were fed regular diet or if they received SMZ/TMP and were symptomatic; and 2) intact EHBD epithelial lining with patent lumen and minimally affected portal tract in SMZ/TMP-*Cxcr2*^-/-^ mice without jaundice (Fig 1C). These data suggested that the superimposition of SMZ/TMP to a *Cxcr2*-deficient state suppresses the biliary atresia phenotype, and were the first indication that potential changes in the microbiome may influence the pathogenesis of disease.

### Effect of SMZ/TMP and RRV infection on bacterial colonization of the neonatal colon

Changes in the bacterial colonization of the neonatal intestinal tract have been linked to abnormalities in the intestinal architecture and the development of the mucosal immune ecosystem [26–28]. To explore whether the exposure to SMZ/TMP affects the intestinal microbiome, DNA samples were obtained from colons of WT and *Cxcr2*^*-/-*^ mice at 3 and 10 days after RRV challenge and submitted for 16S rRNA sequencing. These time points were chosen because they represent the onset of biliary injury (3 days) and allows for the clear distinction between mice with a diseased phenotype and those without jaundice due to the obstruction of EHBD (10 days) [14]. First, to examine the colonization of the neonatal intestinal tract, we investigated the number of mice with ≥ 2500 sequence readouts of bacterial DNA from each colon, a minimum number that reliably reproduces bacterial colonization. At 3 days, we recovered DNA and produced sequence readouts in 40–55% of control neonatal mice (without significant differences between WT and *Cxcr2*^*-/-*^ mice), with adequate DNA reads being recovered from nearly 100% of mice at day 10 (relative ratio [RR] = 3.3 for bacterial colonization; P < 0.0001) (Table 1). Of note, infection of neonatal mice with RRV significantly inhibited the recovery of colons with adequate DNA reads, with colonization documented in ≤ 20% of RRV-infected mice at day 3 (RR = 0.24, or 4.1 times less colonization in RRV infection; P < 0.001) and day 10 (RR = 0.77, 1.3 times less colonization in RRV infection; P < 0.01) when compared to control in WT and *Cxcr2*^*-/-*^ mice (Table 1). Based on the inconsistent recovery of adequate DNA reads in early phases of disease, we focused the analysis of the microbiome at the 10-day time point in experimental and control groups.

**Table 1:** Number of neonatal mice with bacterial colonization in the colon at 3 and 10 days of age.

			Day 3		Day 10	
Treatment	Mouse	Diet	Total	≥ 2500 sequence readouts	Total	≥ 2500 sequence readouts
Control	WT	Regular	11	6 (55%)	16	16 (100%)
		SMZ/TMP	11	4 (36%)	8	8 (100%)
	*Cxcr2*^*-/-*^	Regular	9	4 (44%)	10	9 (90%)
		SMZ/TMP	10	4 (40%)	10	10 (100%)
RRV	WT	Regular	15	3 (20%)	12	12 (100%)
		SMZ/TMP	13	0 (0%)	18	13 (72%)
	*Cxcr2*^*-/-*^	Regular	10	1 (10%)	13	10 (77%)
		SMZ/TMP	9	1 (11%)	14	8 (57%)

### Diverse bacterial and clustering of neonatal mice exposed to SMZ/TMP

**Fig 2:**
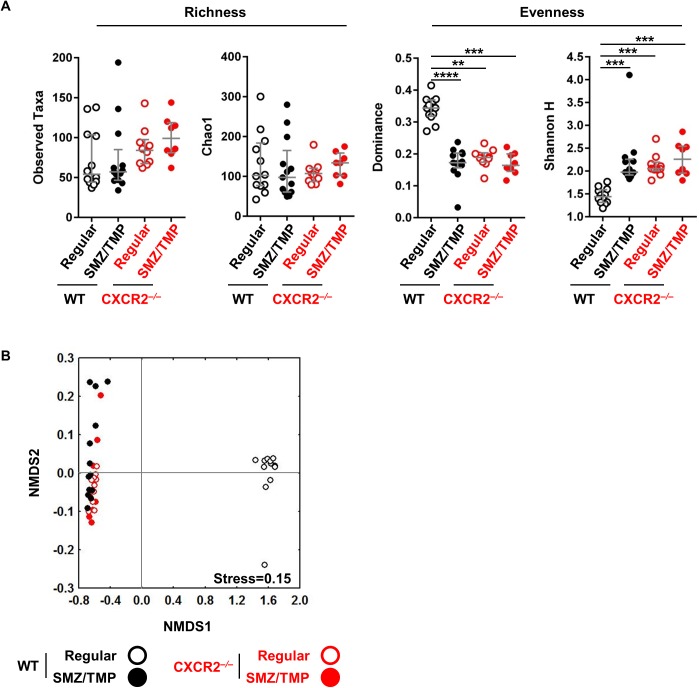
Bacteria diversity in SMZ/TMP-treated WT and Cxcr2^-/-^ mice after RRV infection. (A) Microbial alpha diversity was measured by observed taxa and Chao1 for richness, and by Dominance and Shannon diversity index for evenness (N = 8–13 per group). SMZ/TMP exposure and/or mutation in Cxcr2 significantly increased bacterial diversity without affecting richness. (B) Non-metric dimensional scaling (NMDS) ordinations based upon Bray-Curtis similarity using all OTUs significantly separated regular diet-treated WT mice from all other groups (N = 8–13 per group, P<0.001). Median with interquartile range. *, P<0.05; **, P<0.01; ***, P<0.001; ****, P<0.0001.

Considering previous reports that the neonatal immune system is a critical regulator of the biliary atresia phenotype [29, 30] and that a low diversity of the gut microbiota is linked to intestinal inflammation [9, 31, 32], we measured bacterial α-diversity within each group by calculating the observed number of taxa, Chao1, Dominance and Shannon H. The α-Diversity was not different in saline-control mice regardless of the mouse strain (WT or *Cxcr2*^*-/-*^) or type of diet (regular or SMZ/TMP) (S2A Fig). In contrast, although the total observed and estimated taxa (Chao1) were not different across the groups after RRV infection (Fig 2A), exposure to SMZ/TMP or the loss of *Cxcr2* induced significantly lower Dominance and higher Shannon H index, indicating more diverse bacteria (Fig 2A). Interestingly, the visual ordination of bacterial community using all OTUs (β-diversity) showed that the WT controls on regular diet were the most dissimilar when compared to the other groups (P < 0.001), and all the other groups were closely clustered after RRV infection (Fig 2B), a pattern that was also seen in saline-controls (S2B Fig). Thus, selecting OTUs to distinctly cluster the SMZ/TMP-treated *Cxcr2*^*-/-*^ mice from other groups would be a potential analytical strategy to investigate the link between bacterial profiles and the improved phenotype in neonatal mice infected with RRV.

### Selective bacterial enrichment by SMZ/TMP in neonatal *Cxcr2*^*-/-*^ mice

**Fig 3:**
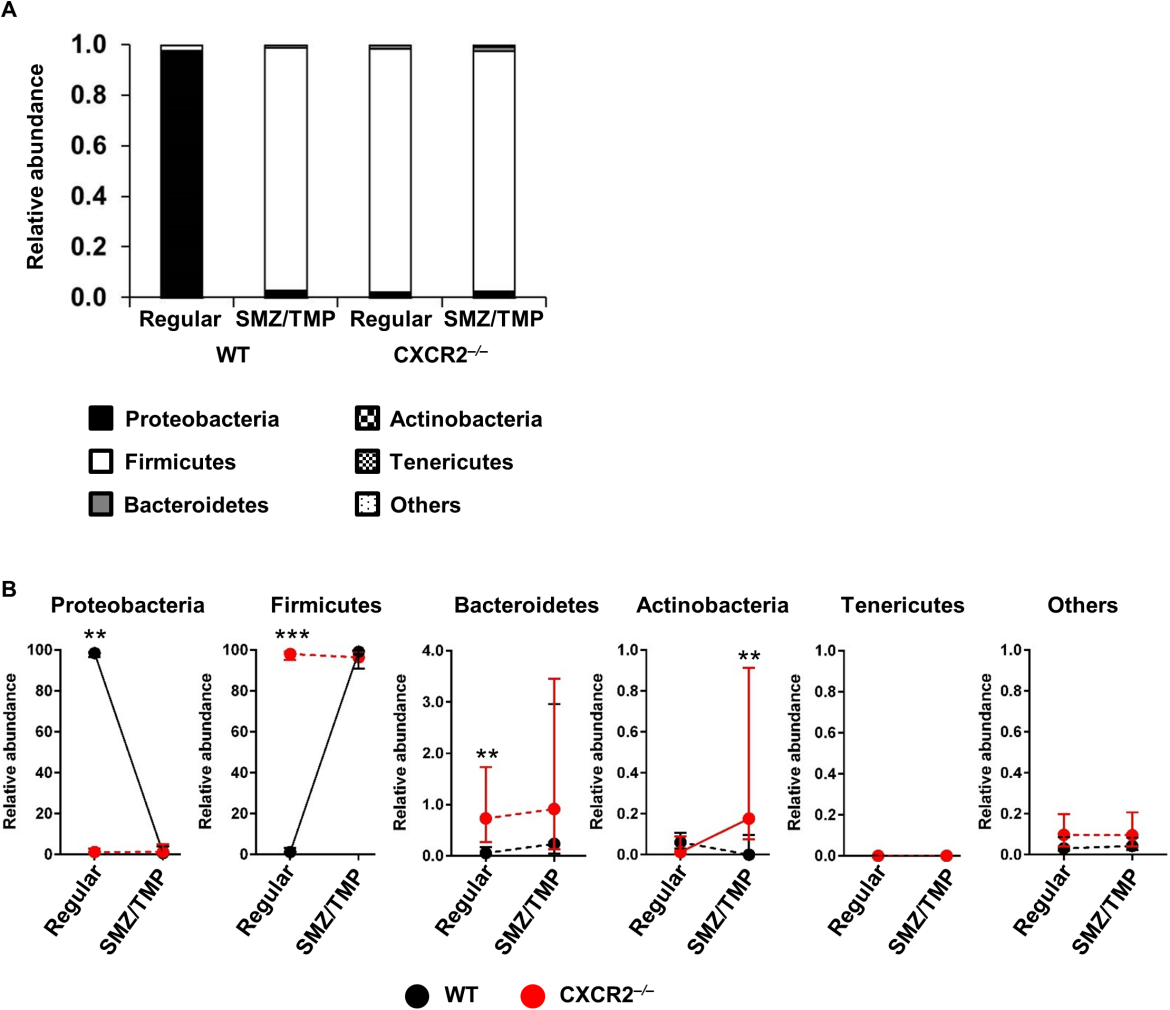
Changes in the intestinal microbiome induced by SMZ/TMP in Cxcr2^-/-^ mice after RRV infection. (A) Phylum analyses show a switch from Proteobacteria to Firmicutes upon exposure to SMZ/TMP or mutation of Cxcr2 (N = 8–13 per group). (B) Colons from the SMZ/TMP-treated Cxcr2^-/-^ mice had less Proteobacteria, more Firmicutes and Actinobacteria (n = 8–13 per group). Median with interquartile ranges in microbiome changes in WT and Cxcr2^-/-^ mice in relation to regular and SMZ/TMP-containing diets (N = 8–13 per group). Solid and dotted lines are defined as statistically significant and non-significant, respectively, between regular and SMZ/TMP diet in each mouse type. *P<0.05; **P<0.01; ***P<0.001; ****P<0.0001.

The analysis of the microbiome at the phylum level has been used to characterize various diseases in early life [9]. Analyzing the phyla in all mice infected with RRV, we found that WT colons had a predominance of *Proteobacteria* in WT-regular diet group, with a small population of *Firmicutes* (Fig 3A). This relationship was reversed by exposing mice to SMZ/TMP or by the loss of *Cxcr2* (Fig 3A). Within the group of *Cxcr2*^*-/-*^ mice, SMZ/TMP increased the abundance of *Actinobacteria* (0.07% in regular diet vs. 0.73% in SMZ/TMP treatment, P < 0.01; Fig 3B).

### Enrichment of bacterial genus by SMZ/TMP in neonatal *Cxcr2*^*-/-*^ mice

**Fig 4:**
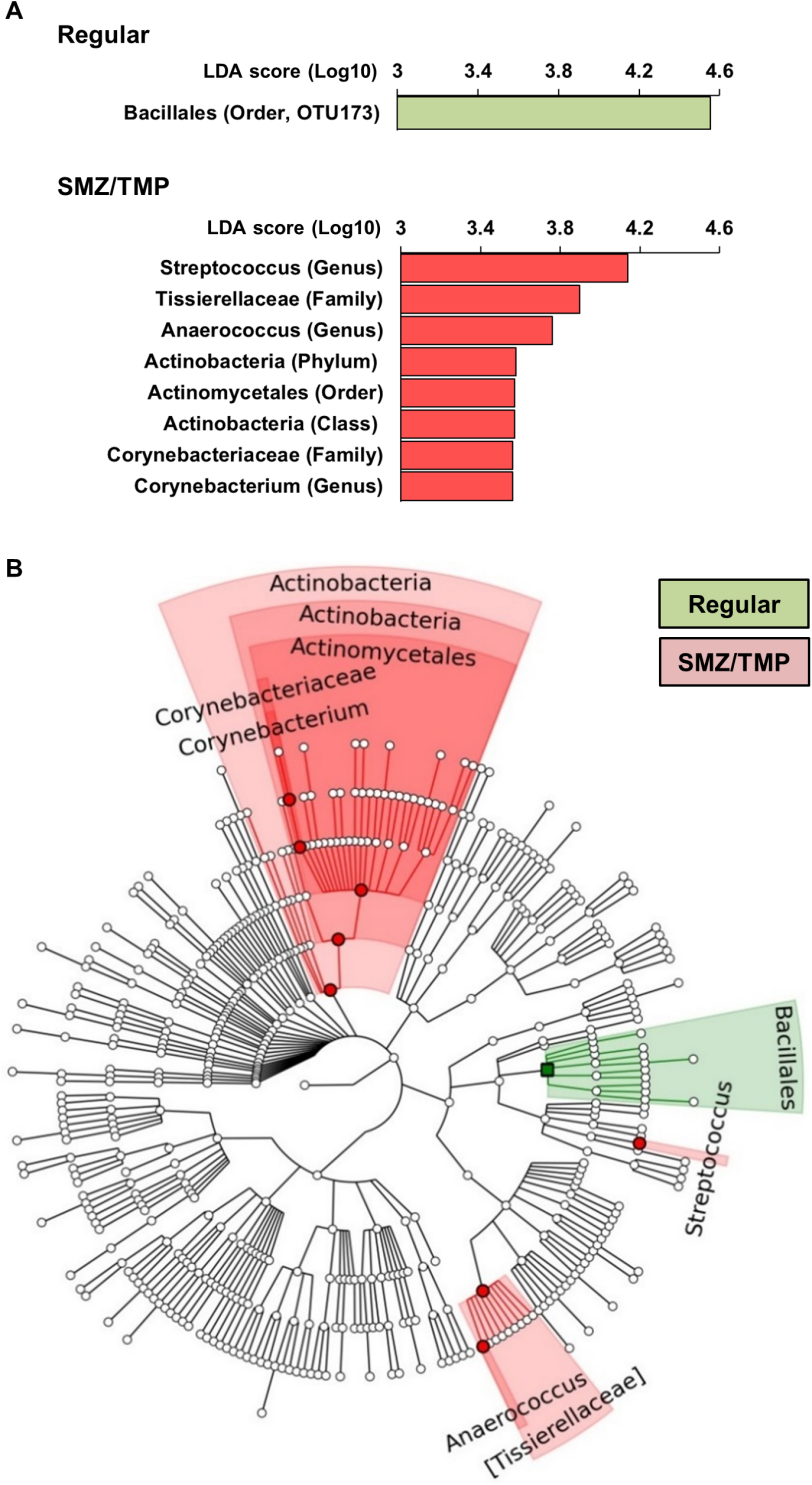
Gut-Microbiome changes induced by SMZ/TMP in Cxcr2^-/-^ mice after RRV infection. (A) Bacterial taxa representing significantly different abundances between the regular (green) and SMZ/TMP (red) diets in Cxcr2^-/-^ mice according to their LDA scores. (B) Cladograms were generated on the basis of the LDA scores, showing significantly different abundance between regular and SMZ/TMP diet. Green-highlighted regions are indicative of taxa more abundant in regular diet, and red-highlighted regions, more abundant in SMZ/TMP diet (N = 8–13 per group).

**Fig 5:**
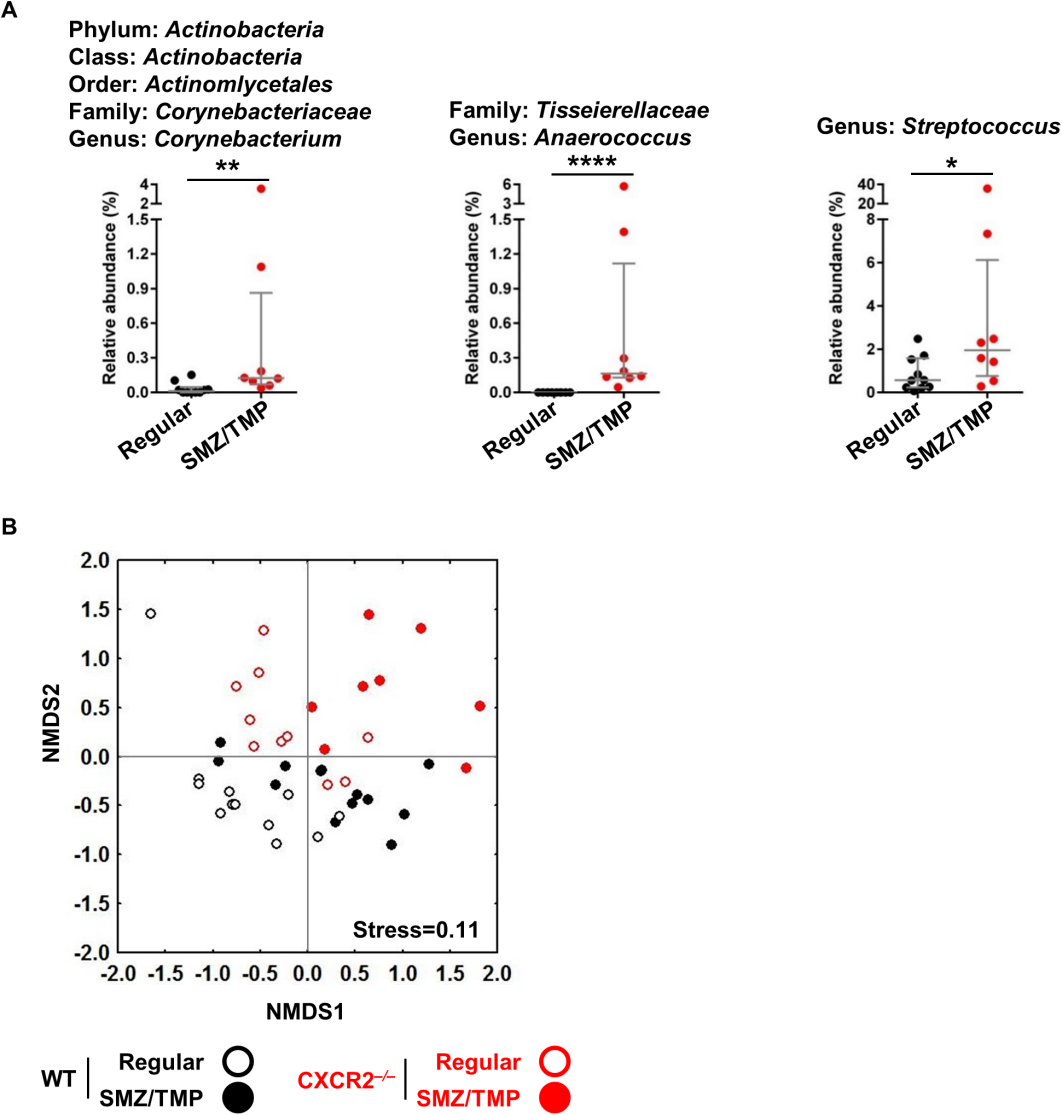
Signature bacteria strongly associated with improvement of BA-disease phenotypes. (A) Corynebacterium, Anaerococcus and Streptococcus were recognized as signature bacteria in Cxcr2^-/-^ mice, significantly more abundant in SMZ/TMP than in regular diet (N = 8–10 per group). (B) Non-metric dimensional scaling (NMDS) ordinations based upon Bray-Curtis similarity using the three candidate bacteria OTUs significantly separated SMZ/TMP-Cxcr2^-/-^ pups from all other groups (N = 8–13 per group, P<0.001). Median with interquartile range. *, P>0.05; **, P<0.01; ***, P<0.001; ****, P<0.0001.

To more directly investigate which changes in the microbiome are linked to the improved phenotype induced by SMZ/TMP, we focused on the analysis of the OTU data from *Cxcr2*^*-/-*^ mice, which had a general predominance of *Firmicutes* and *Actinobacteria* (Fig 3A and 3B). Using linear discriminant analysis (LDA) effect size (LEfSe), we found an enrichment of *Bacillales* order (LDA score of 4.5) on a regular diet whereas treatment with SMZ/TMP increased *Streptococcus* genus, *Tissierellaceae*, *Actinobacteria* and *Corynebacteriaceae* (LDA scores of 4.1, 3.8, 3.5, and 3.5, respectively; Fig 4A). Among these, *Tissierellaceae*, *Actinobacteria* and *Corynebacteriaceae* were represented at ≥ 2 levels in a cladogram (Fig 4B), with variable population of the genera in individual mice, with the exception of *Anaerococcus* that was not detected in all *Cxcr2*^*-/-*^ mice of the regular diet group (Fig 5A).

Only the SMZ/TMP-treated Cxcr2^*-/-*^ mice showed improvement in the BA-disease phenotypes (Fig 1), but clustering the SMZ/TMP-treated Cxcr2^*-/-*^ mice using all OTUs (Fig 2B) was not successful. However, when only the three bacterial candidates were used, the SMZ/TMP-treated Cxcr2^*-/-*^ mice were distinctively clustered in the NMDS, which significantly differed from all the other groups (Fig 5B). Thus, the results suggest that the three genera, *Corynebacterium*, *Anaerococcus* and *Streptococcus* are the candidate bacteria that are strongly associated with improvement of the BA-disease phenotypes.

### *Anaerococcus lactolyticus* most clearly differentiates high from low survivability in neonatal *Cxcr2*^*-/-*^ mice

**Fig 6:**
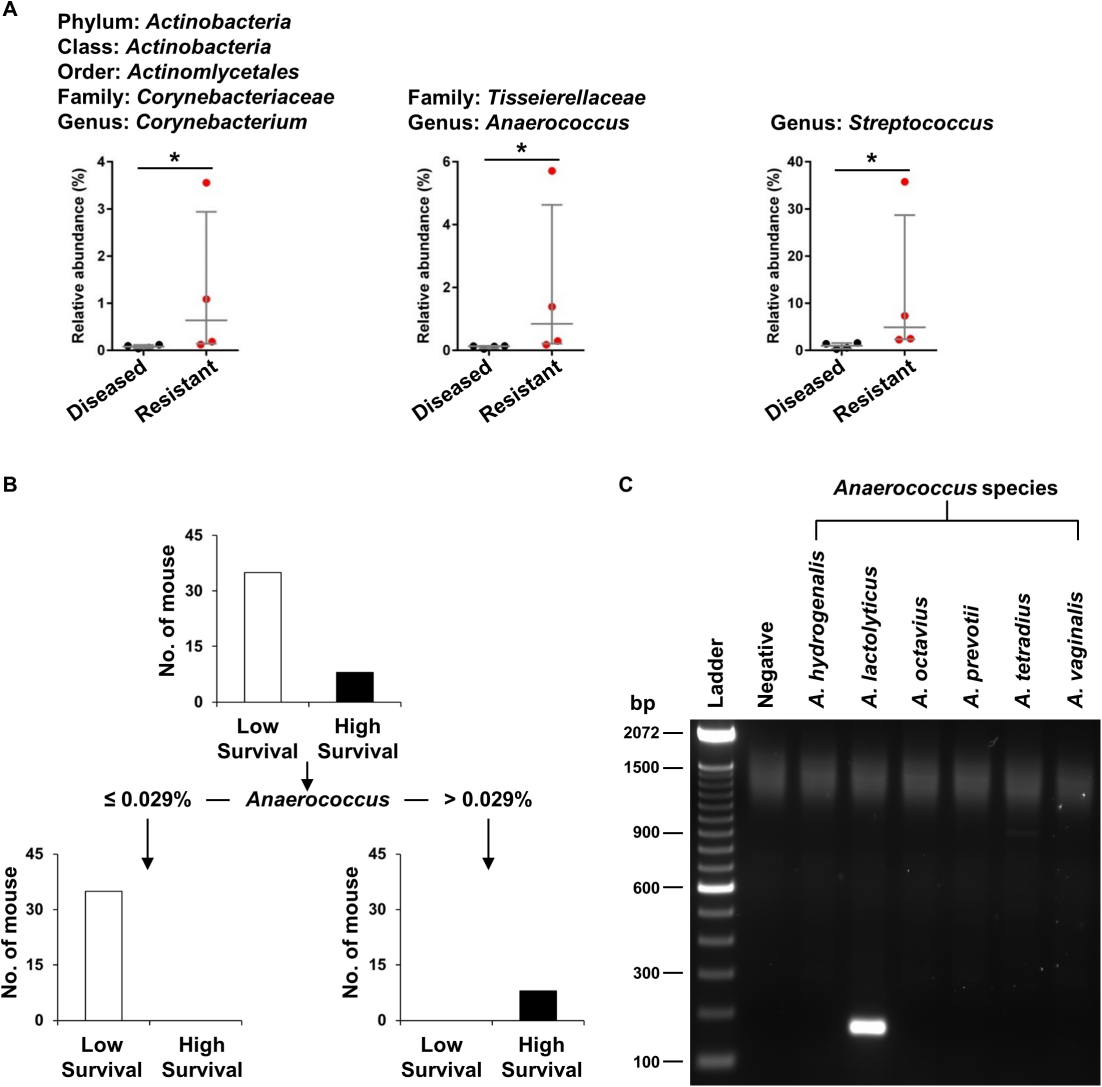
Microbiome changes in Cxcr2^-/-^ mice with symptoms (diseased) and without symptoms (resistant) 14 days after RRV infection. (A) Relative abundance of three genera in the colon of diseased and resistant Cxcr2^-/-^ mice exposed to SMZ/TMP diet (N = 4 per group). (B) CART analysis of all three genera identifies Anaerococcus genus most clearly differentiating the high from low survivability (N = 43). (C) Nested-PCR shows only A. lactolyticus in colons of Cxcr2^-/-^ mice exposed to SMZ/TMP (N = 8). Median with interquartile range. *, P<0.05; **, P<0.01; ***, P<0.001; ****, P<0.0001.

To more precisely link specific microbiome changes with the biliary atresia phenotype, we compared OTU data in the group of *Cxcr2*^*-/-*^ mice without symptoms after RRV infection (here called “resistant”) with the group showing full phenotypic features of biliary atresia (here called “diseased”). Our hypothesis was that the genera are more abundant in colons from resistant than diseased littermates. Analyzing OTU using the Mann-Whitney test, we found significant populations of all genera in resistant mice (P< 0.05, Fig 6A). Next, we used CART analyses to determine the genera that differentiates between the resistant and diseased outcomes. Using this approach, only *Anaerococcus* clearly differentiates the survival outcomes, with mice that contain >0.029% *Anaerococcus* of total OTU segregating exclusively with survival (Fig 6B). Last, to examine the *Anaerococcus* genus at the species level, we used nested PCR with primer sets to detect 6 known species. Among these, only *Anaerococcus lactolyticus* (amplicon size ~150 bp) was detected from the resistant *Cxcr2*^*-/-*^ mice (Fig 6C).

### Decreased mRNA for chemokines and cytokines related to Cxcr2

**Fig 7:**
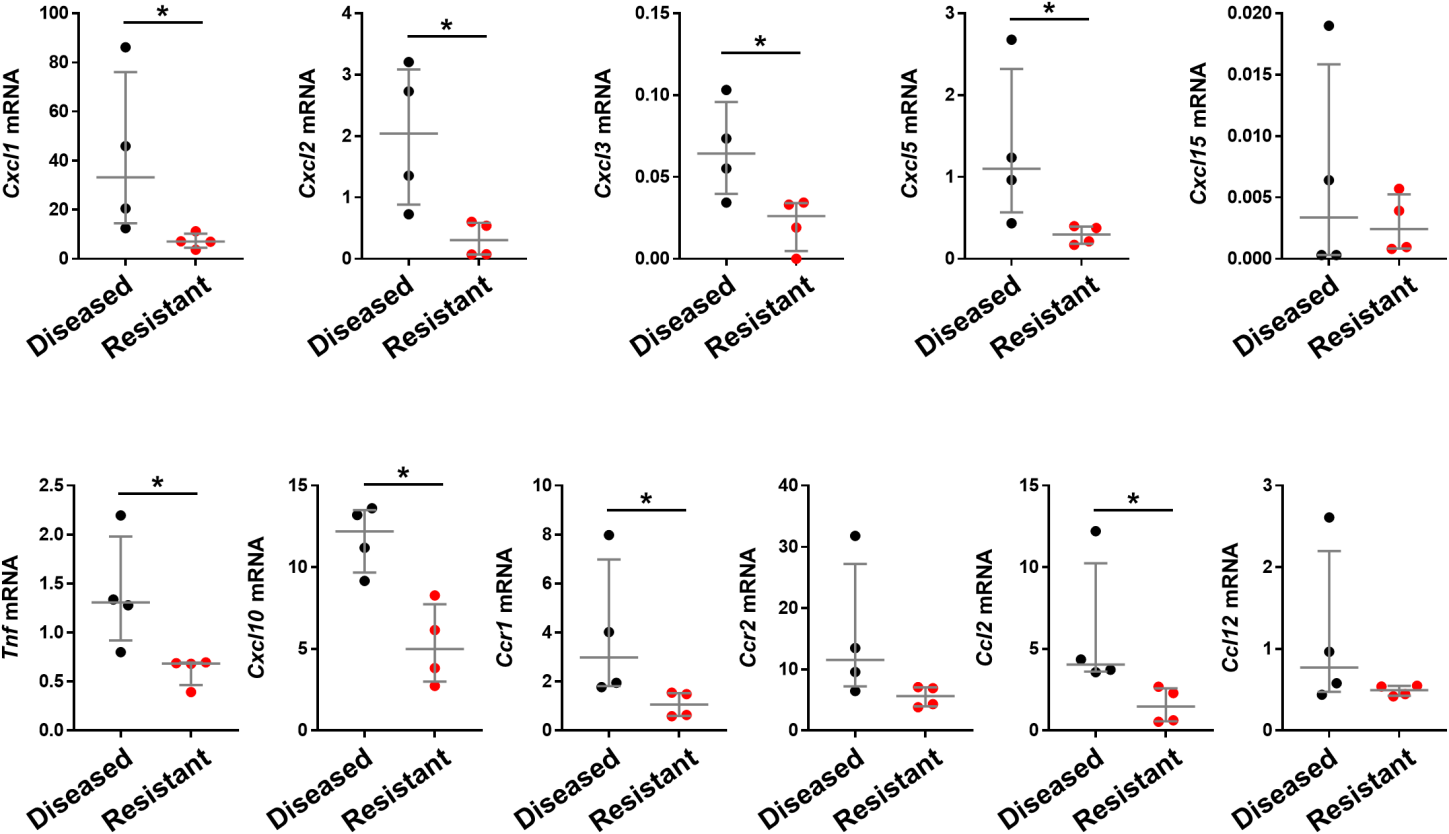
Hepatic expression for Cxcr2-related chemokines and cytokines. mRNA expression levels quantified by real-time PCR using RNA from livers of neonatal Cxcr2^-/-^ mice with the biliary atresia phenotype (diseased) or asymptomatic (resistant) mice 14 days after RRV infection. mRNA is expressed as a ratio to Gapdh (N = 4 per group). Median with interquartile range. * P<0.05.

To determine whether the improved clinical phenotype is linked to a decrease tissue expression of inflammation-related genes, we quantified the liver mRNA expression of 11 chemokines and cytokines that are either linked to Cxcr2 signaling and/or reported to be effector of biliary injury. Data from real-time PCR showed a significant down-regulation of mRNAs for *Cxcl1*, *Cxcl2*, *Cxcl3*, *Cxcl5*, *Cxcl10*, *Ccr1*, *Ccl2*, and *Tnf* (but not for *Cxcl15*, *Ccr2*, or *Ccl12*) in livers of mice with the resistant phenotype when compared to the diseased phenotype (Fig 7).

## Discussion

We found that the exposure of *Cxcr2*^*-/-*^ mice to SMZ/TMP suppressed cholestasis, decreased the incidence of biliary obstruction, and improved survival. Analyzing the microbiome for a potential effect of SMZ/TMP on the colonization of the neonatal intestinal tract and its relationship to the improved outcome, we found a switch to a *Firmicutes*-rich microbial signature in the neonatal colon. Notably, the inactivation of *Cxcr2* alone induced a similar switch, with the additional exposure of *Cxcr2*^*-/-*^ mice to SMZ/TMP enriching the bacterial signature with *Corynebacterium*, *Anaerococcus* and *Streptococcus* in those mice with improved cholestasis and survival. Among these bacteria, the population of *Anaerococcus* (specifically *Anaerococcus lactolyticus*) above 0.029% of the overall taxonomic units was significantly predictive of survival. These data suggest that the changes in the development of commensal bacteria in neonatal mice may play a role in the mechanisms of bile duct injury and phenotype determination in experimental biliary atresia.

The exposure of pregnant and lactating adult BALB/c mice to SMZ/TMP switched the microbiome of the offspring from *Proteobacteria*-rich to a predominance with *Firmicutes* and induced a survival benefit over controls (not receiving the antibiotic). Although the improvement in outcome was modest (only 19%) in wild-type mice, it served as the first evidence that additional factors may offer synergy and provide a greater benefit against biliary injury after RRV infection. In this regard, the similar switch in the microbiome signature induced by the inactivation of *Cxcr2* raised the possibility that a subpopulation of commensals induced by the additive effects of SMZ/TMP could be linked to the substantial improvement in cholestasis and survival observed in this group of mice. In keeping with this possibility, we found a consistent enrichment with *Anaerococcus* in *Cxcr2*^*-/-*^ mice resistant to the biliary atresia phenotype when compared to susceptible (diseased) mice.

Although little is known about the mechanisms by which Cxcr2 (CXCR2 in humans) or its ligands (Cxcl-1, 2, 5; IL-8 in humans) modulate the phenotypes of hepatobiliary diseases via changes in the intestinal microbiome, the Cxrc2-dependent circuit plays an important role in neutrophil-migration into infected or ulcerated site of an intestinal tract [33, 34]. Further, activated neutrophils have been shown to stimulate colonic epithelial cells to produce antimicrobial peptides (AMPs) such as regenerating islet-derived protein III beta (regIIIβ) and S100A8 (calgranulin A) [35]. Neutrophils themselves produce AMPs (defensin, bactericidal/permeability increasing protein, lysozyme, lactoferrin, lipocalin-2 and cathelicidin) [36], some of which may modulate bactericidal activities by binding to the outer membrane lipoprotein and by changing membrane permeability [37, 38]. Consistent with these biological scenarios, we found a suppression of several genes encoding cytokines and chemokines related to Cxcr2. These data form a rationale for future studies to explore how these molecular circuits and factors may individually or collectively form a biological link between the microbiome in the developing neonate and the pathogenesis of tissue injury in experimental biliary atresia.

The microbiome has not undergone a comprehensive analysis in infants with biliary atresia, but a previous report showed that only 2.4% of the stool cultures from infants with biliary atresia contained bifidobacteria in contrast to 75% in healthy infants [39]. A recent study investigated the frequency of ascending cholangitis in groups of infants receiving oral *Lactobacillus casei rhamnosus*, neomycin, or no antibiotic to prevent recurrent cholangitis after hepatoportoenterostomy [40]. Ascending cholangitis was diagnosed in 20% of subjects receiving *Lactobacillus casei rhamnosus* or neomycin, which was much lower than the 80% frequency in the no-intervention group. In our experimental approach, the *Lactobacillus* genus was more abundant in BALB/c mice receiving SMZ/TMP, but not linked to an improved phenotype in these or *Cxcr2*^*-/-*^ mice. We recognize the substantial differences between the murine and human intestinal microbiomes, and do not yet have human data to make direct comparisons. This limitation notwithstanding, the findings of specific links between the microbiome, decreased inflammation, and resistance to the biliary atresia phenotype raise an important paradigm for a potential role of changes in commensal bacteria in the pathogenesis of the disease in humans. Interestingly, our proposed relationship of the *Anaerococcus* genus with the penetrance of the disease phenotype experimentally may be applicable to humans based on the presence of the genus as a gut-commensal bacteria detected in 4-month old infants [41]. Although there may be an important overlap in microbiome changes between mice and infants with biliary atresia, it is important to await human data and minimize premature implication of specific commensals in the pathogenesis of the disease in humans.

The statistical link of *Anaerococcus lactolyticus* with the group of *Cxcr2*^*-/-*^ mice having improved cholestasis and outcome points to a potential role of this bacteria in ameliorating the outcome of biliary atresia. One of the major metabolic end-products from *A*. *lactolyticus* cultured with peptone/yeast extract/glucose medium is butyrate [42], a short-chain fatty acid that has been demonstrated to modulate inflammatory responses. For example, in a mouse model of experimental colitis, the concentration of butyrate was significantly increased in the lumen of colons that contained a greater abundance of IL-10^+^Foxp3^+^ cells, resulting in amelioration of colitis in mice fed with butyrate-supplemented diet [43]. In humans, the addition of butyrate to the culture condition of lamina propria and peripheral blood mononuclear cells from patients with Crohn’s disease stimulated with LPS prevented the translocation of NF-κB p65 from cytoplasm to nucleus, and consequently produced less amount of TNF-α, IL-6 and IL-1β [44]. Formal studies are required to generate a greater insight into how *A*. *lactolyticus* and Cxcr2 signaling regulate the mechanisms that suppress biliary injury in neonatal mice.

In summary, maternally administered SMZ/TMP vertically modified the colonization of the neonatal intestines by commensal bacteria in the mouse model. Among the microbiome changes, the population of *A*. *lactolyticus* in mice carrying the genetic inactivation of *Cxcr2* was associated with a resistance to the experimental phenotype of biliary atresia, expressed as decreased cholestasis, biliary injury, and improved long-term survival. Although we do not yet know how *A*. *lactolyticus* or a related metabolite (such as butyrate) directly relates to Cxcr2 signaling to suppress the tissue injury, our data add a new biological dimension to the proposed pathogenic mechanisms of biliary atresia. These data also form the rationale for future human-based studies to investigate the microbiome profiles at diagnosis and following hepatoportoenterostomy. The potential influence of the intestinal microbiome on short- and long-term outcomes is particularly intriguing because the newly created roux-en-Y loop creates a new environment where the potential colonization with predominant bacterial species may be an important factor for infectious complications and/or for progression to from the liver inflammation to end-stage cirrhosis in children with biliary atresia.

## Supporting information

S1 Fig Pipeline workflow for 16S rRNA pre-processing step. A LONI pipeline, composed of PANDAseq, QIIME and USEARCH was used for all 16S rRNA pre-processing step to obtain OTU tables.
(TIF)

S2 Fig Bacterial diversity in SMZ/TMP-treated WT and *Cxcr2*^*-/-*^ mice receiving saline as controls. (A) Microbial alpha diversity was measured by observed taxa and Chao1 for richness, and by Dominance and Shannon diversity index for evenness (n = 8–13 per group). SMZ/TMP exposure and/or mutation in *Cxcr2* significantly increased bacterial diversity without affecting richness. (B) Non-metric dimensional scaling (NMDS) ordinations based upon Bray-Curtis similarity using all OTUs significantly separated regular diet-treated WT mice from all other groups (n = 8–16 per group, P<0.001). Median with interquartile range. *, P<0.05; **, P<0.01; ***, P<0.001; ****, P<0.0001.
(TIF)

S1 Table Oligonucleotide primer sets (forward and reverse) used in real-time PCR for hepatic gene-expression analysis. (DOCX)

S2 Table Oligonucleotide primers for 16s rRNA and *Anaerococcus* species. Species detection by PCR used the combination of the following primers: #5 and 4 for *A*. *hydrogenalis* (amplicon size ~ 400 bp), #6 and 4 for *A*. *lactolyticus* (~ 150 bp), #7 and 4 for *A*. *octavius* (~ 760 bp), #8 and 4 for *A*. *prevotii* (~ 400 bp), #9 and 3 for *A*. *tetradius* (~ 150 bp), and #10 and 4 for *A*. *vaginalis* (~760 bp).
(DOCX)
