# Supplementary figures and images for: Cxcr2 signaling and the microbiome suppress inflammation, bile duct injury, and the phenotype of experimental biliary atresia
Source: PLoS One. 2017 Aug 1;12(8):e0182089. doi: 10.1371/journal.pone.0182089 (PMC5538677; doi:10.1371/journal.pone.0182089)

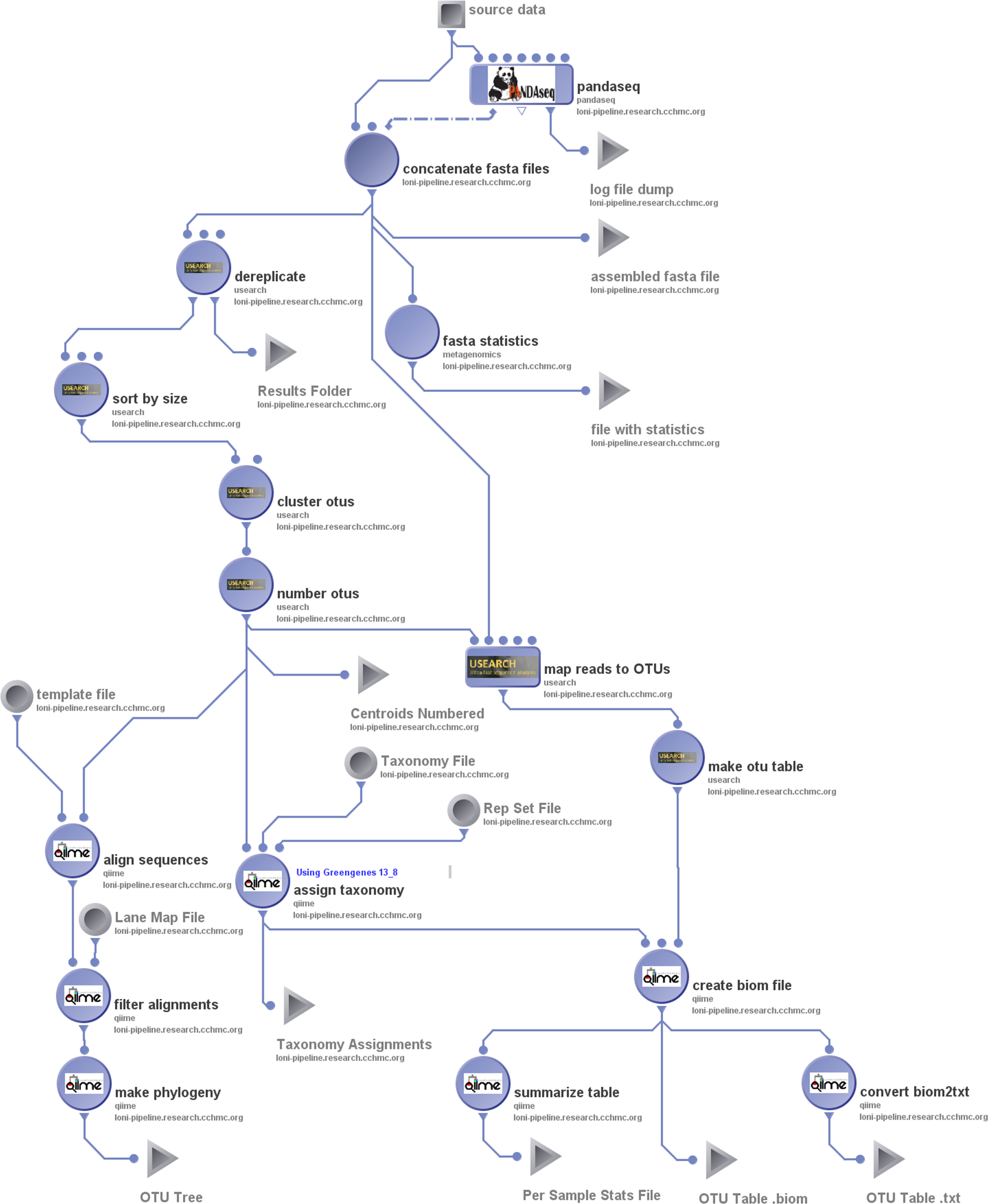

Supplement: S1 Fig — A LONI pipeline, composed of PANDAseq, QIIME and USEARCH was used for all 16S rRNA pre-processing step to obtain OTU tables. (TIF) [file pone.0182089.s001.tif]

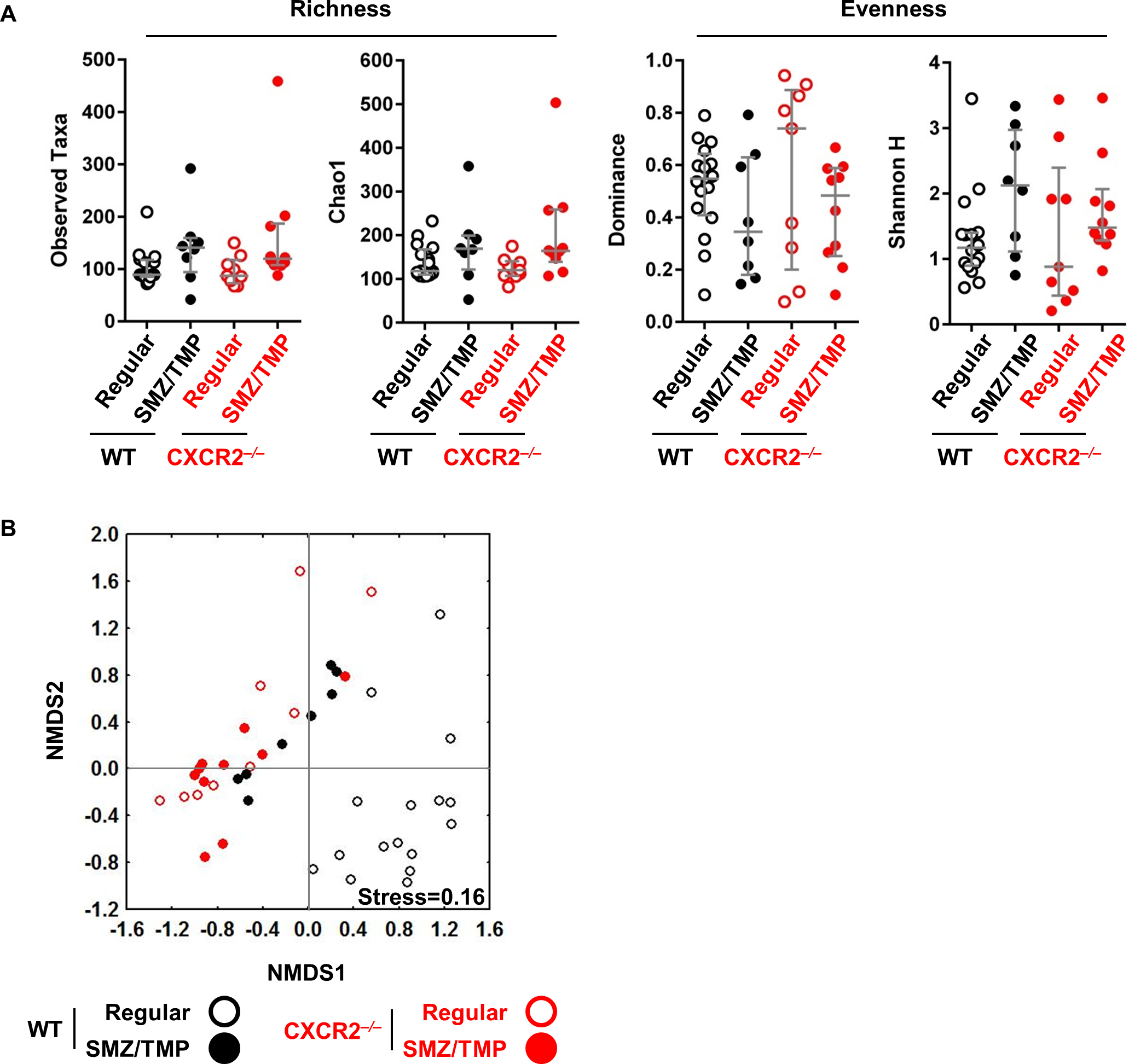

Supplement: S2 Fig — (A) Microbial alpha diversity was measured by observed taxa and Chao1 for richness, and by Dominance and Shannon diversity index for evenness (n = 8–13 per group). SMZ/TMP exposure and/or mutation in Cxcr2 significantly increased bacterial diversity without affecting richness. (B) Non-metric dimensional scaling (NMDS) ordinations based upon Bray-Curtis similarity using all OTUs significantly separated regular diet-treated WT mice from all other groups (n = 8–16 per group, P<0.001). Median with interquartile range. *, P<0.05; **, P<0.01; ***, P<0.001; ****, P<0.0001. (TIF) [file pone.0182089.s002.tif]
