# Supplementary material for: Cxcr2 signaling and the microbiome suppress inflammation, bile duct injury, and the phenotype of experimental biliary atresia
Source: PLoS One. 2017 Aug 1;12(8):e0182089. doi: 10.1371/journal.pone.0182089 (PMC5538677; doi:10.1371/journal.pone.0182089)
Supplement: S1 Table — (DOCX) [file pone.0182089.s003.docx]

**S1 Table. Oligonucleotide primer sets (Forward and Reverse) used in real-time PCR for hepatic gene-expression analysis**

| Target gene | Sequence (5’→3’) |
| --- | --- |
| *Cxcl1* | Forward: ACCGAAGTCATAGCCACACTC  Reverse: TGGGGACACCTTTTAGCATC |
| *Cxcl2* | Forward: TGAACAAAGGCAAGGCTAACTG  Reverse: AAGTGAACTCTCAGACAGCGAGG |
| *Cxcl3* | Forward: CATCCAGAGCTTGACGGTGA  Reverse: TTGGGGGTTGAGGCAAACTT |
| *Cxcl5* | Forward: TGGGCAGTGACAAAAAGAAAGC  Reverse: AAATCCGTGGGTGGAGAGAATC |
| *Cxcl15* | Forward: TGATGCTCCATGGGTGAAGG  Reverse: CAGAAGCTTCATTGCCGGTG |
| *Tnf* | Forward: ATGGCCTCCCTCTCATCAGT  Reverse: TTGGTGGTTTGCTACGACGT |
| *Cxcl10* | Forward: TTTCTGCCTCATCCTGCTGG  Reverse: CATTCTCACTGGCCCGTCAT |
| *Ccr1* | Forward: TCTTAGCTTCCATGCCTGCC  Reverse: TCCACTGCTTCAGGCTCTTG |
| *Ccr2* | Forward: TGAGCCTGATCCTGCCTCTA  Reverse: AAAGATGAGCCTCACAGCCC |
| *Ccl2* | Forward: CAGCCAGATGCAGTTAACGC  Reverse: GCTGCTGGTGATCCTCTTGT |
| *Ccl12* | Forward: TCAGGTATTGGCTGGACC  Reverse: GGCTGCTTGTGATTCTCC |
| *Gapdh* | Forward: TGGTTTGACAATGAATACGGCTAC  Reverse: GGTGGGTGGTCCAAGGTTTC |
