# Supplementary material for: Cxcr2 signaling and the microbiome suppress inflammation, bile duct injury, and the phenotype of experimental biliary atresia
Source: PLoS One. 2017 Aug 1;12(8):e0182089. doi: 10.1371/journal.pone.0182089 (PMC5538677; doi:10.1371/journal.pone.0182089)
Supplement: S2 Table — Species detection by PCR used the combination of the following primers: #5 and 4 for A. hydrogenalis (amplicon size ~ 400 bp), #6 and 4 for A. lactolyticus (~ 150 bp), #7 and 4 for A. octavius (~ 760 bp), #8 and 4 for A. prevotii (~ 400 bp), #9 and 3 for A. tetradius (~ 150 bp), and #10 and 4 for A. vaginalis (~760 bp). (DOCX) [file pone.0182089.s004.docx]

**S2 Table. Oligonucleotide primers for 16s rRNA and *Anaerococcus* species**

Primer

Sequence (5’→3’)

8F

1492R

341B

*A. hydrogenalis*

*A. lactolyticus*

*A. octavius*

*A. prevotii*

*A. tetradius*

*A. vaginalis*

ATTTCCTTACGACTTCGGTC

CCGTCAATTCMTTTRAGTTT

CAGAGCAGCTAAACAGCGATGTCA

AACTGGAGACCTTGAGTAATGG

GGTCTAGAGATAGACTCTTA

TGACATAAACTCTTCGCAT

CCGTCAATTCMTTTRAGTTT

AGAGTTTGATCCTGGCTCAG

GGTTACCTTGTTACGACTT

1392B

ACGGGCGGTGTGTAC

No.

1

2

3

4

5

6

7

8

9

10
